# Supplementary material for: Regulation of multiple dimeric states of E-cadherin by adhesion activating antibodies revealed through Cryo-EM and X-ray crystallography
Source: PNAS Nexus. 2022 Aug 19;1(4):pgac163. doi: 10.1093/pnasnexus/pgac163 (PMC9491697; doi:10.1093/pnasnexus/pgac163)
Supplement: pgac163_Supplemental_File [file pgac163_supplemental_file.pdf]

Supplemental Data

Supplementary methods; Supplementary Tables S1-S4, and Supplementary figures S1-S11

Regulation of multiple dimeric states of E-cadherin by adhesion activating antibodies revealed through Cryo-EM and X-ray crystallography

Allison Maker, Madison Bolejack, Leslayann Schecterson, Brad Hammerson, Bart Staker, Peter Myler, Barry M Gumbiner

Supplementary Methods

Bio-Layer interferometry

BLI kinetics assays were performed on an Octet Red96 at 23°C, shaking at 1000 rpm. Protein was diluted in kinetics buffer: 50 mM Tris pH 8.0, 150 mM NaCl, 3mM $CaCl_2$, 0.25 mg/mL BSA, 0.005% Tween-20. Ni-NTA sensors (ForteBio) were equilibrated for 60 seconds, then E-cadherin EC1-5-TwinStrep-8His was loaded onto the sensor for 180 seconds, followed by another 60 second baseline. Sensors were then immersed into a 1:3 dilution series of anti-E-cadherin Fabs in kinetics buffer until they reached desired concentrations, then dipped into empty kinetics buffer to determine off-rates. ForteBio data analysis software was used to calculate kinetics parameters such as k_{on}, k_{off}, and K_D using a 1:1 binding global fit model. Assays were repeated at least twice with different Fab preparations to ensure consistent results. For 19A11 and 46H7, both ficin-cleaved untagged Fabs and TwinStrep tagged Fabs were tested; both showed similar affinities regardless of presence of tag (Extended Data Figure 4).

Analytical size exclusion chromatography

2.5 uM hE-cadherin EC1-5 TwinStrep were incubated with 3.2x molar excess Fab (2x by mass) at 4°C for ~16 hours. Mixtures were then injected into a Superose 6 10/300 column. For analysis, elution times were multiplied by the 0.5 mL/min flow rate to calculate elution volume in mL. Fractions were run on 5-20% SDS-PAGE gels to examine protein composition of each peak.

Colo205 Activation Assay

The Colo205 activation assay was performed as described previously[17]. Briefly, Colo205 cells were densely plated on 96-well plates precoated with 0.1μg/mL rat-tail collagen (Sigma-Aldrich) overnight and treated with activating concentrations of Fabs for 5 hours. Activation was determined by the extent of a morphological change from round cells with distinct borders to a compact epithelial appearance and loss of obvious cell borders.

	19A11	46H7	59D2	67G8	66E8
K_D calc	8.05E-09	9.46E-07	1.19E-12	9.59E-09	9.01E-08
	5.10E-09	2.58E-07	2.42E-12	4.56E-09	9.65E-08
	6.71E-09	3.24E-07	1.81E-12	1.09E-08	9.18E-08
	6.42E-09		1.88E-12	9.52E-09	
Average	6.57E-09	5.09E-07	1.83E-12	8.64E-09	9.28E-08
Std dev	1.21-09	3.80E-07	5.03E-13	2.79E-09	3.34E-09

Table S1. Individual measurements and kinetic-based calculations of Fab affinity.

PDB	Protein
**3Q2V**	Mouse E-cadherin EC1-5
**2O72**	Human E-cadherin EC1-2
**6CXY**	Human E-cadherin EC1-2/19A11 Fab
**7STZ**	Human E-cadherin EC1-5/19A11 Fab
**6VEL**	Human E-cadherin EC1-2/66E8 Fab

Table S2. PDBs created or referenced in this study

	hEC1-2/66E8	hEC1-5/19A11
Wavelength	0.9787	0.9787
Resolution range	46.54 - 2.65 (2.745 - 2.65)	49.54 – 2.95 (3.055 – 2.95)
Space group	P 31 2 1	P 1 21 1
Unit cell	142.17 142.17 90.32 90 90 120	85.34 131.62 201.76 90 100.861 90
Total reflections	269829 (27242)	685577 (50776)
Unique reflections	30878 (3042)	92265 (6783)
Multiplicity	8.7 (9.0)	7.4 (7.5)
Completeness (%)	99.9 (99.9)	99.9 (100.0)
Mean I/sigma(I)	26.01 (3.86)	14.25 (3.20)
R-merge	0.0620 (0.562)	0.118 (0.631)
R-meas	0.066 (0.596)	0.127 (0.678)
CC1/2	0.999 (0.934)	0.997 (0.897)
Reflections used in refinement	30872 (3040)	92206 (9197)
Reflections used for R-free	1542 (171)	1996 (200)
R-work	0.1889 (0.2568)	0.1822 (0.2731)
R-free	0.2380 (0.3134)	0.2079 (0.2974)
CC(work)	0.931 (0.877)	0.937 (0.856)
CC(free)	0.889 (0.873)	0.950 (0.812)
Number of non-hydrogen atoms	4890	14690
macromolecules	4729	13868
ligands	33	288

solvent	128	534
Protein residues	630	1837
RMS(bonds)	0.004	0.003
RMS(angles)	0.68	0.55
Ramachandran favored (%)	96.94	95.80
Ramachandran allowed (%)	2.74	3.92
Ramachandran outliers (%)	0.32	0.28
Rotamer outliers (%)	2.49	1.87
Clashscore	5.44	4.16
Average B-factor	72.07	79.85

Table S3. X-ray data collection and refinement statistics.

Cryo-EM data collection and processing statistics				
**Sample**	**Full-length E-cadherin-catenin complex + 19A11**	**Full-length E-cadherin-catenin complex + 46H7**	**Full-length E-cadherin + 59D2**	**Full-length E-cadherin + 67G8**
**Data collection**				
Microscope	Titan Krios	Titan Krios	Titan Krios	Titan Krios
Voltage (kV)	300	300	300	300
Magnification	130000x	130000x	105000x	105000x
Detector	Gatan K2	Gatan K2	Gatan K3	Gatan K3
Data collection software	Leginon	Leginon	Leginon	Leginon
Electron exposure (e^-/Å^2)	40	40	47	64
Defocus Range (μm)	-1 - -2.5	-1 - -2.5	-1 - -2.5	-1 - -2.5
Pixel size (Å)	0.525	0.525	0.84	0.42
**Data processing**				
Number of micrographs	1823	2004	3805	1655
Final particle images	99879	67509	331400	97712
Symmetry imposed	C1	C1	C1	C1
Map resolution (Å) 0.143 FSC threshold	4.85	4.75	6.24	5.55

Table S4. Cryo-EM data collection, reconstruction, and refinement.

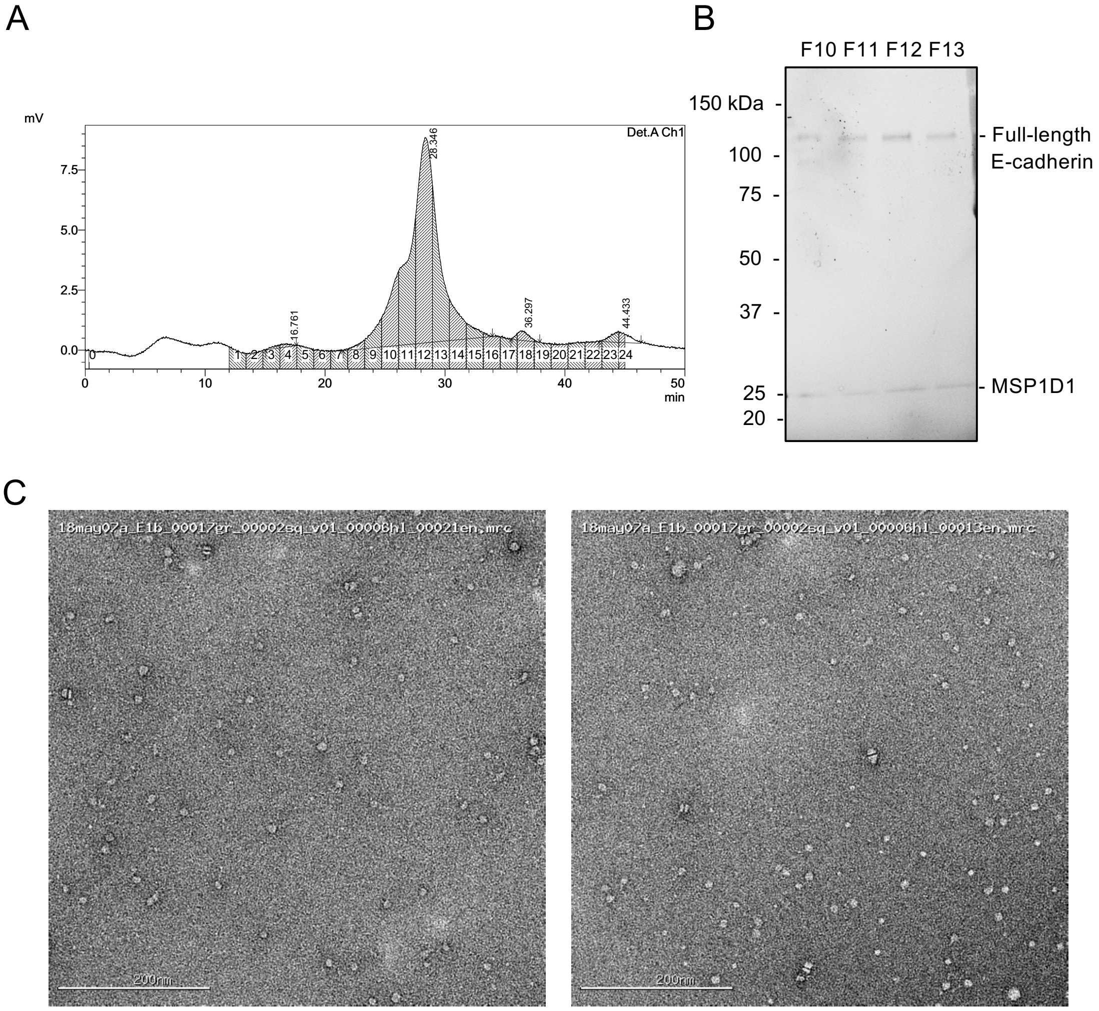

Figure S1. Full-length E-cadherin is embedded in MSP1D1 nanodiscs. (A) SEC chromatogram of FL-hE-cadherin nanodiscs. (B) SDS-PAGE gel of SEC peak fractions indicating presence of E-cadherin and MSP1D1 membrane scaffold protein (C) Negative stain EM micrographs of E-cadherin nanodiscs. Minor stacking is evident from the calcium content in the buffer.

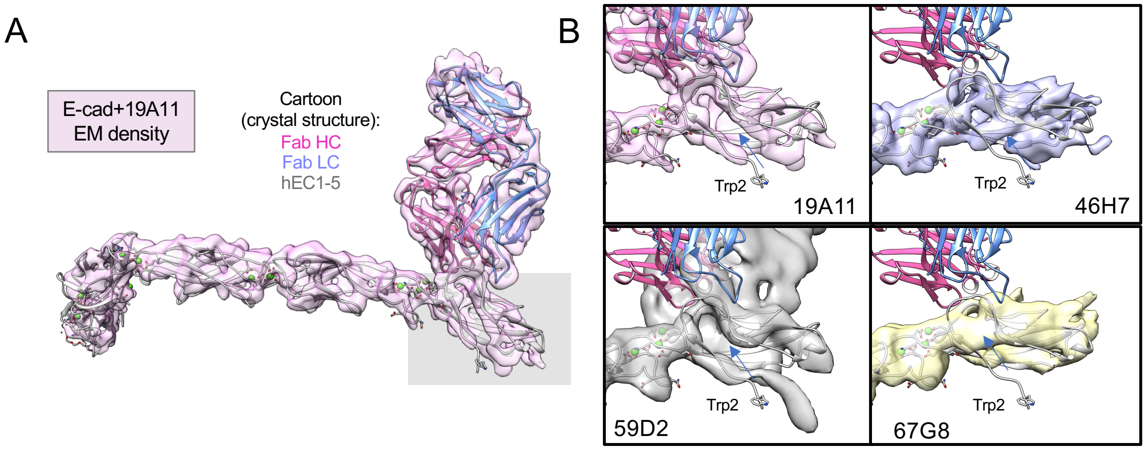

Figure S2. EM reconstructions of activating and non-activating Fabs have variations in EC1 density. (A) Overlay of monomeric EM reconstruction and crystal structure of hEC1-5/19A11. Grey box highlights general EC1 region examined in (B). Closeups of EC1 with each Fab bound, overlaid with EC1-5/19A11 structure to indicate location of beta strand and Trp2. The arrow indicates the location of the hydrophobic pocket.

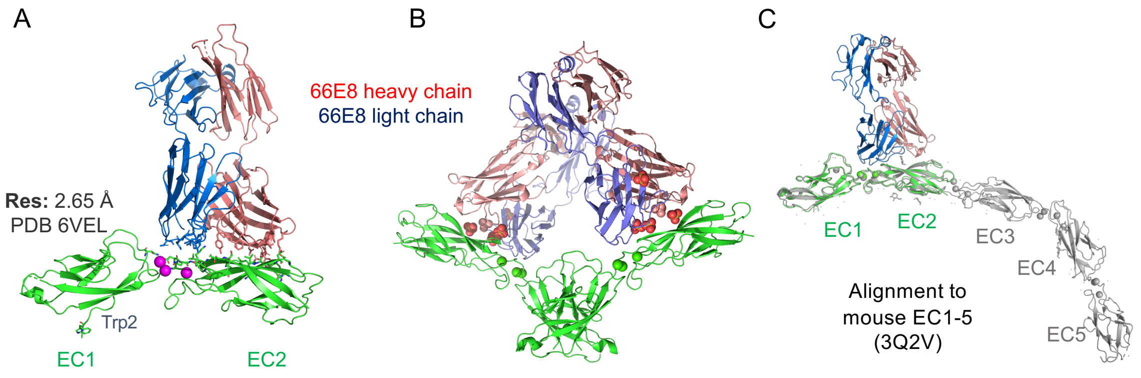

Figure S3. Crystal structure of hEC1-2/66E8 activating Fab. (A) Asymmetric unit of crystal structure indicating Fab epitope in EC2 and the EC1-2 Ca binding site. (B) Strand-swap dimer seen in crystal expansion (C) Overlay with mouse EC1-5 PDB indicating epitope location in full ectodomain.

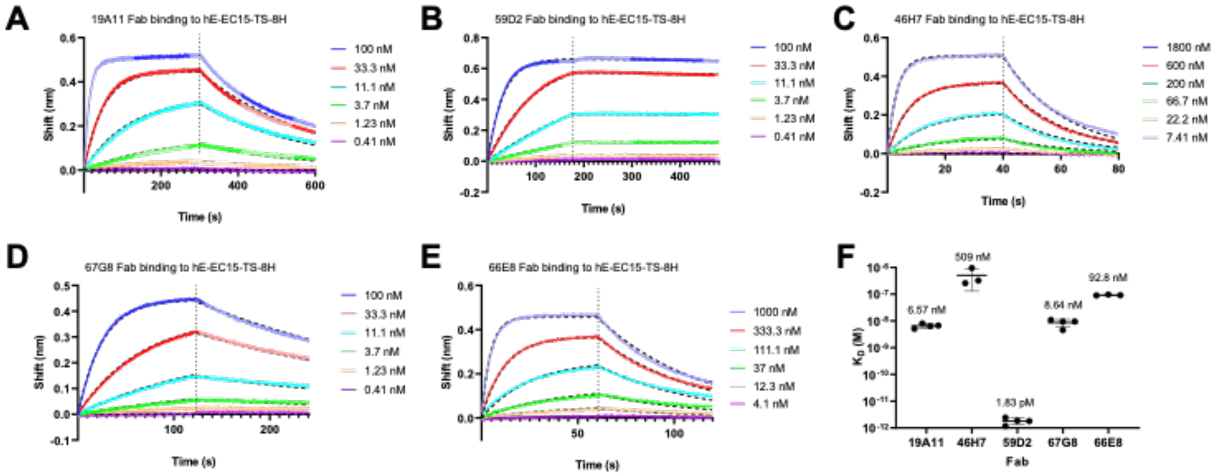

Figure S4. BLI kinetics of Fabs binding to hEC1-5-TS-8His. (A) 19A11 Fab binding curve. (B) 59D2 Fab binding curve. (C) 46H7 binding curve. (D) 67G8 binding curve.(E) 66E8 binding curve (F) summary of individual measurements. Mean K_D labeled for each Fab.

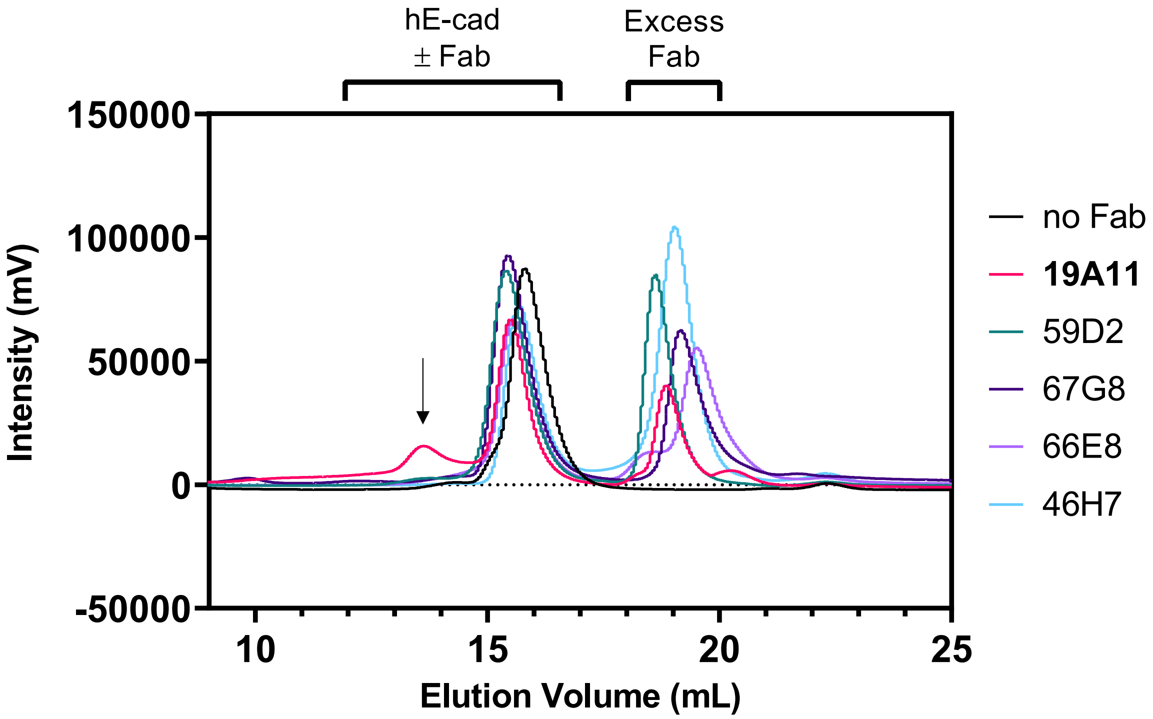

Figure S5. SEC of all recombinant functional antibodies bound to hEC1-5 shows that 19A11 is prominent in its formation of a hEC1-5 dimer peak.

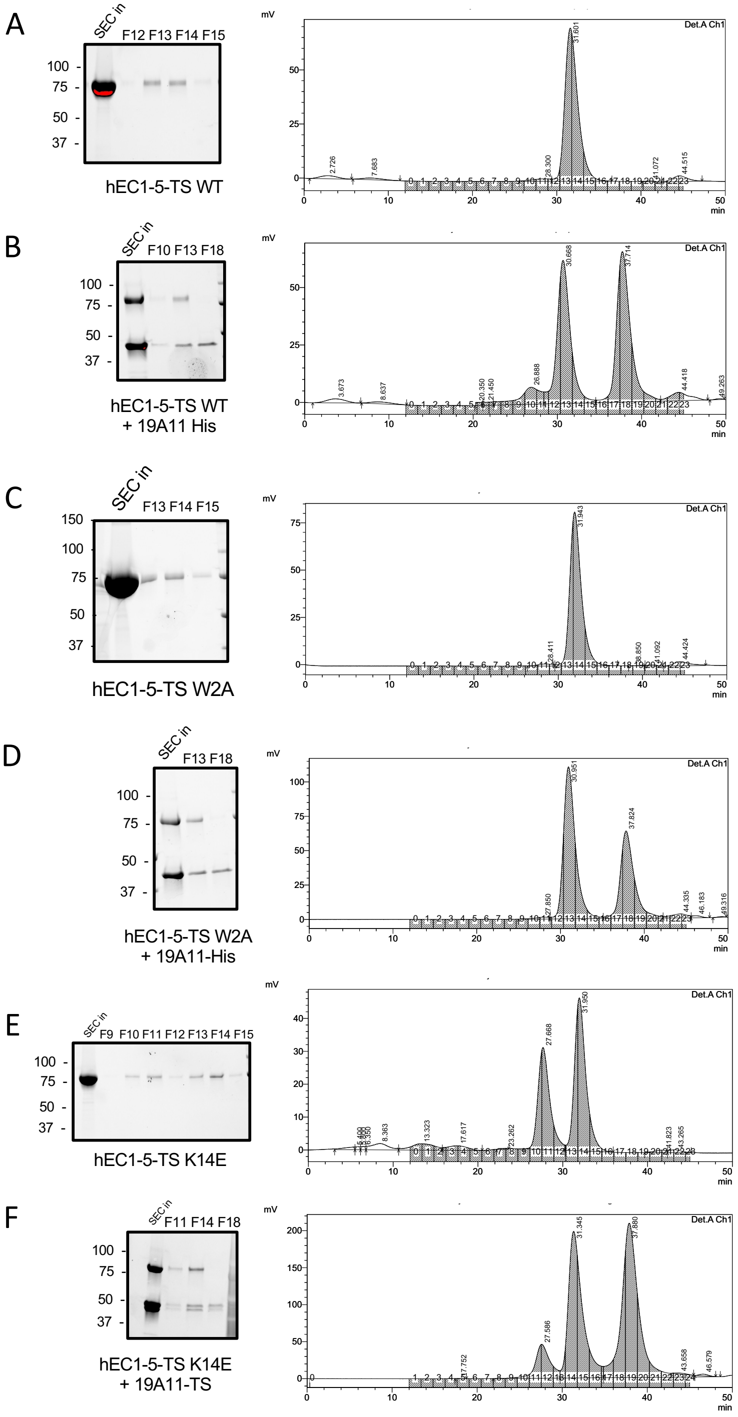

Figure S6. Individual raw SEC chromatograms and gels of fractions of human E-cadherin ectodomain Twin Strep (hEC1-5-TS) mutants bound to 19A11 Fab. Fraction numbers indicated at the bottom of each chromatogram. F(#) = Fraction # in gel legend. (A) hEC1-5-TS WT alone (B) hEC1-5 TS WT mixed with and excess of 19A11 Fab. (C) hEC1-5 TS W2A strand-swap deficient mutant. (D) hEC1-5 TS W2A mixed with and excess of 19A11 Fab. (E) hEC1-5 TS K14E X-dimer blocking mutant alone. (F) hEC1-5-TS K14E mixed with an excess of 19A11 Fab.

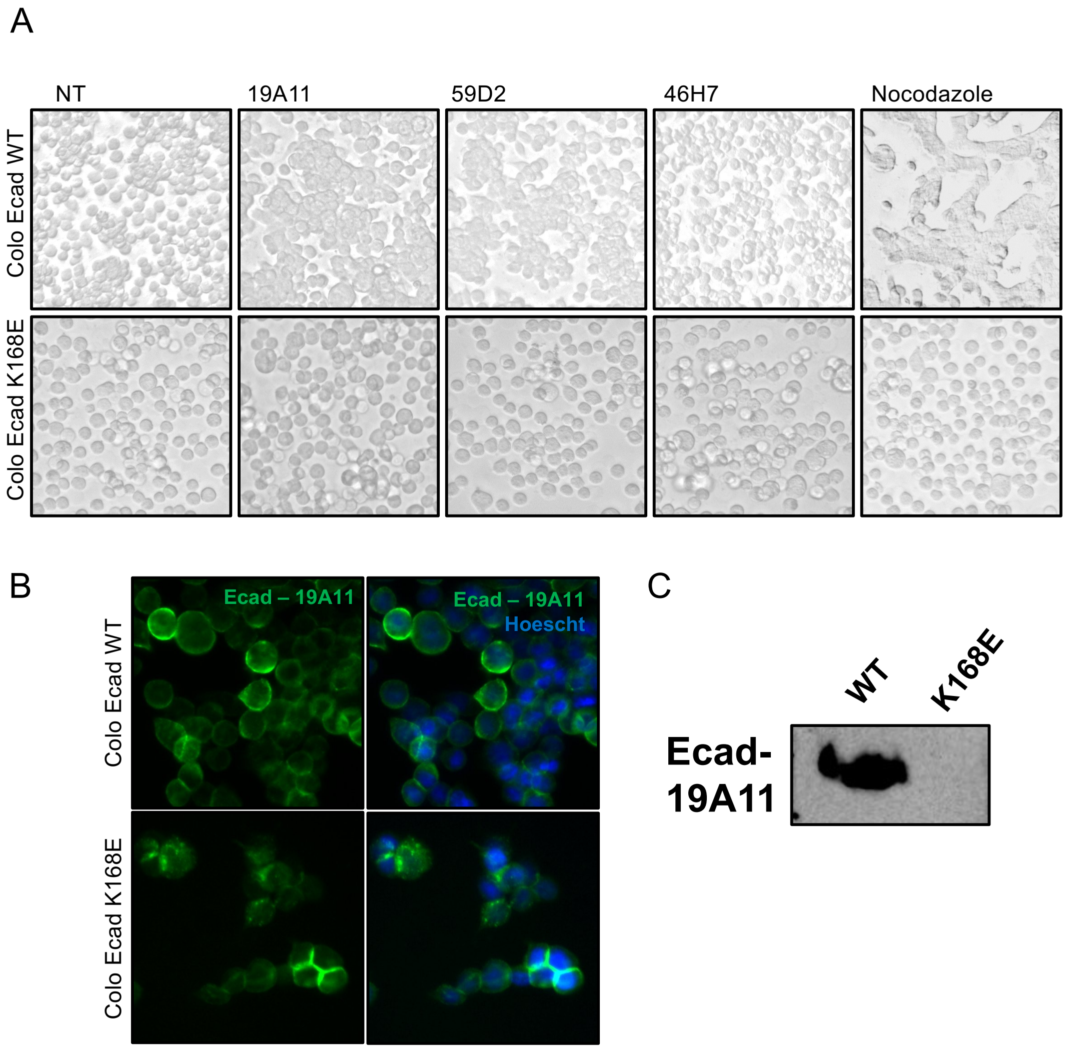

Figure S7. Colo205 activation with K14E/K168E E-cadherin is not rescued by 19A11. (A) Colo205 activation assay of WT E-cadherin expressing cells and K168E E-cadherin cells with full mAb treatment. NT = no treatment. (B) Immunofluorescence staining of WT and K168E E-cadherin Colo205 cells with 19A11 full mAb. (C) Western blot of Colo205 cell lysates expressing either WT E-cadherin or K168E.

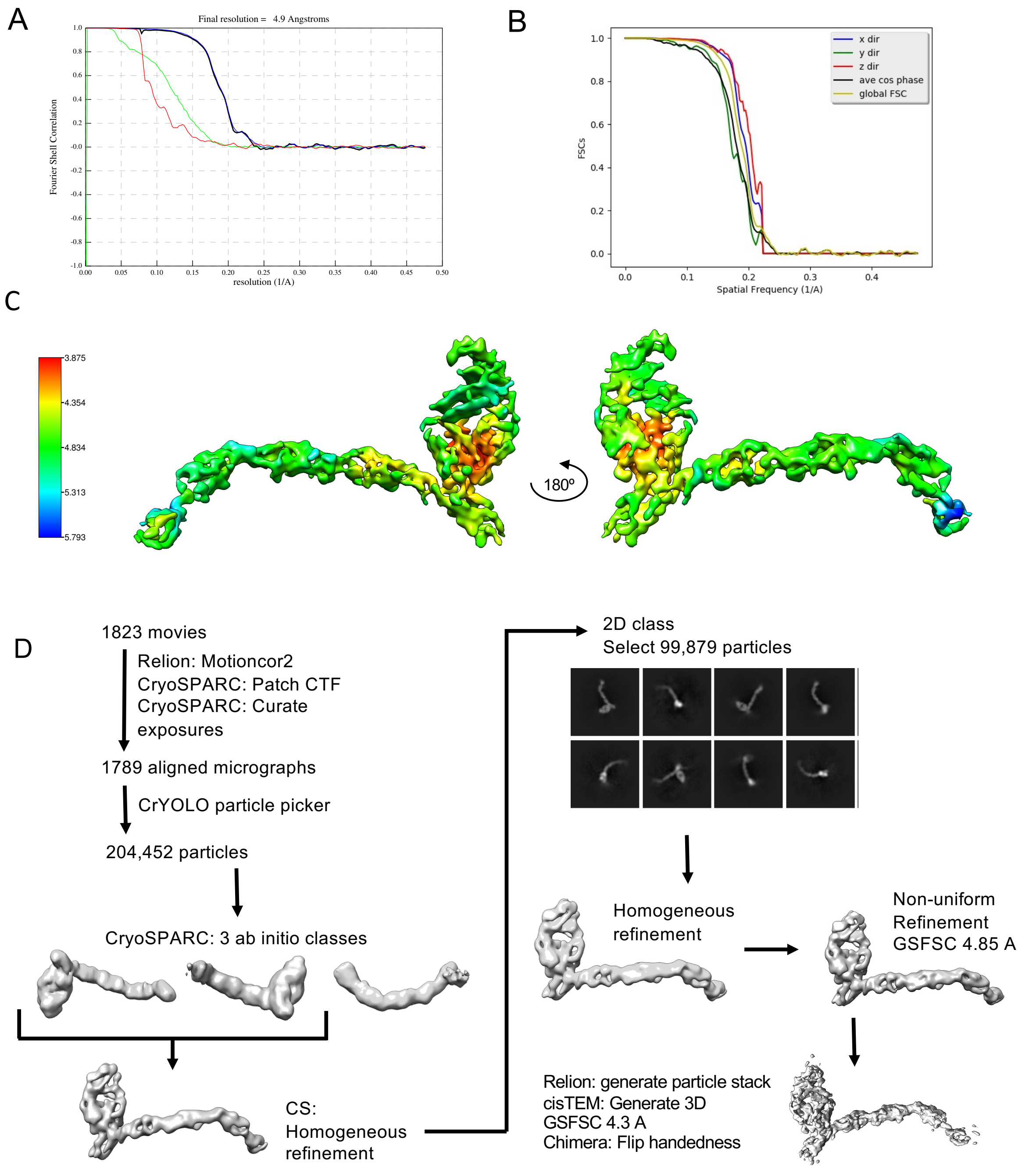

Figure S8. Cryo-EM characterization of FL-hE-cadherin + 19A11Fab. **(A)** Relion Gold-Standard 0.143 FSC resolution of final reconstruction. **(B)** Directional resolution of map calculated by 3DFSC server. Calculated sphericity: 0.966. **(C)** Local resolution estimation over sharpened final 3D reconstruction. **(D)** Data processing pipeline toward final reconstruction.

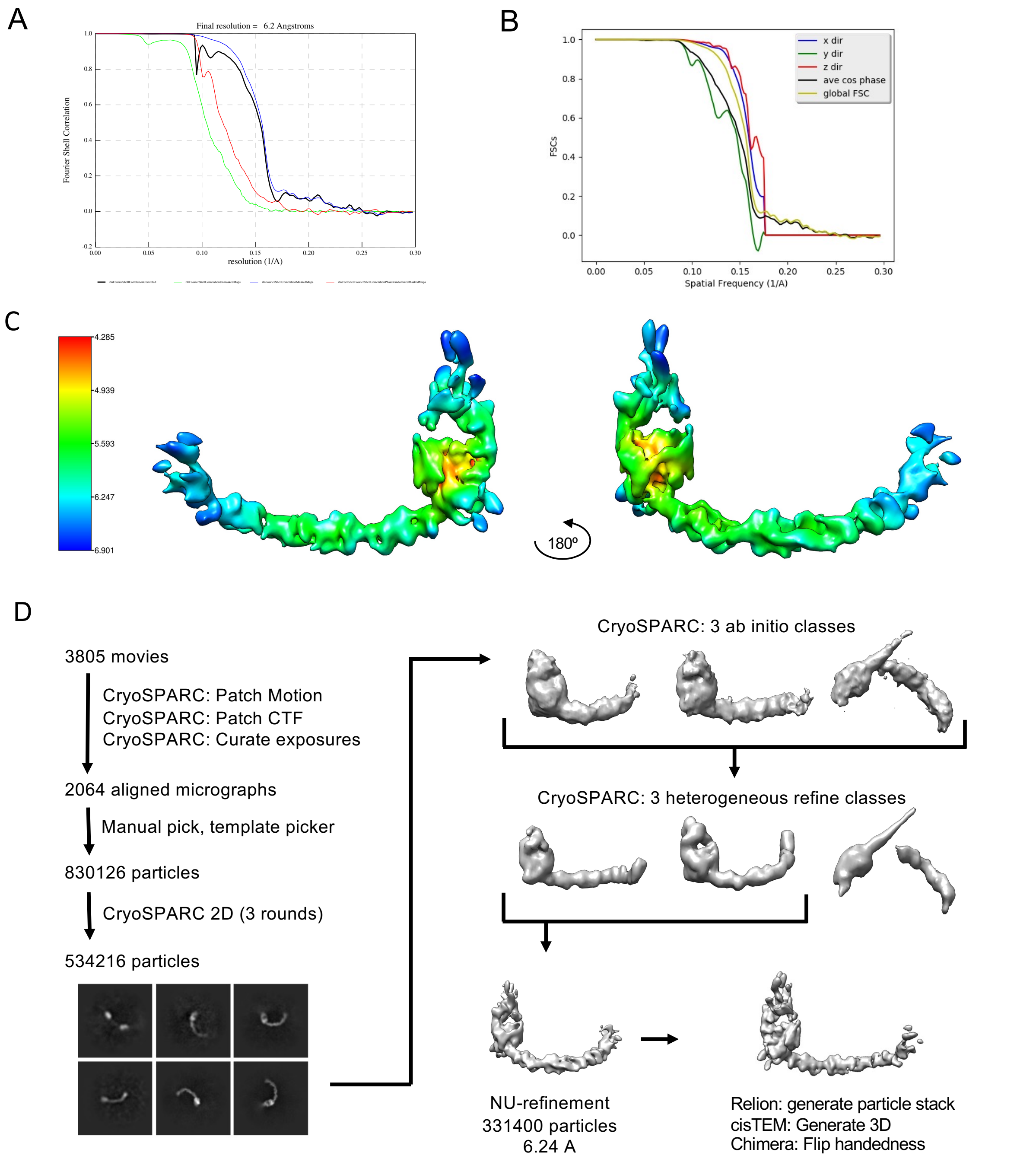

Figure S9. Cryo-EM characterization of FL-hE-cadherin + 59D2Fab. (**A)** Relion Gold-Standard 0.143 FSC resolution of final reconstruction. **(B)** Directional resolution of map calculated by 3DFSC server. Calculated sphericity: 0.973. **(C)** Local resolution estimation over sharpened final 3D reconstruction. **(D)** Data processing pipeline toward final reconstruction.

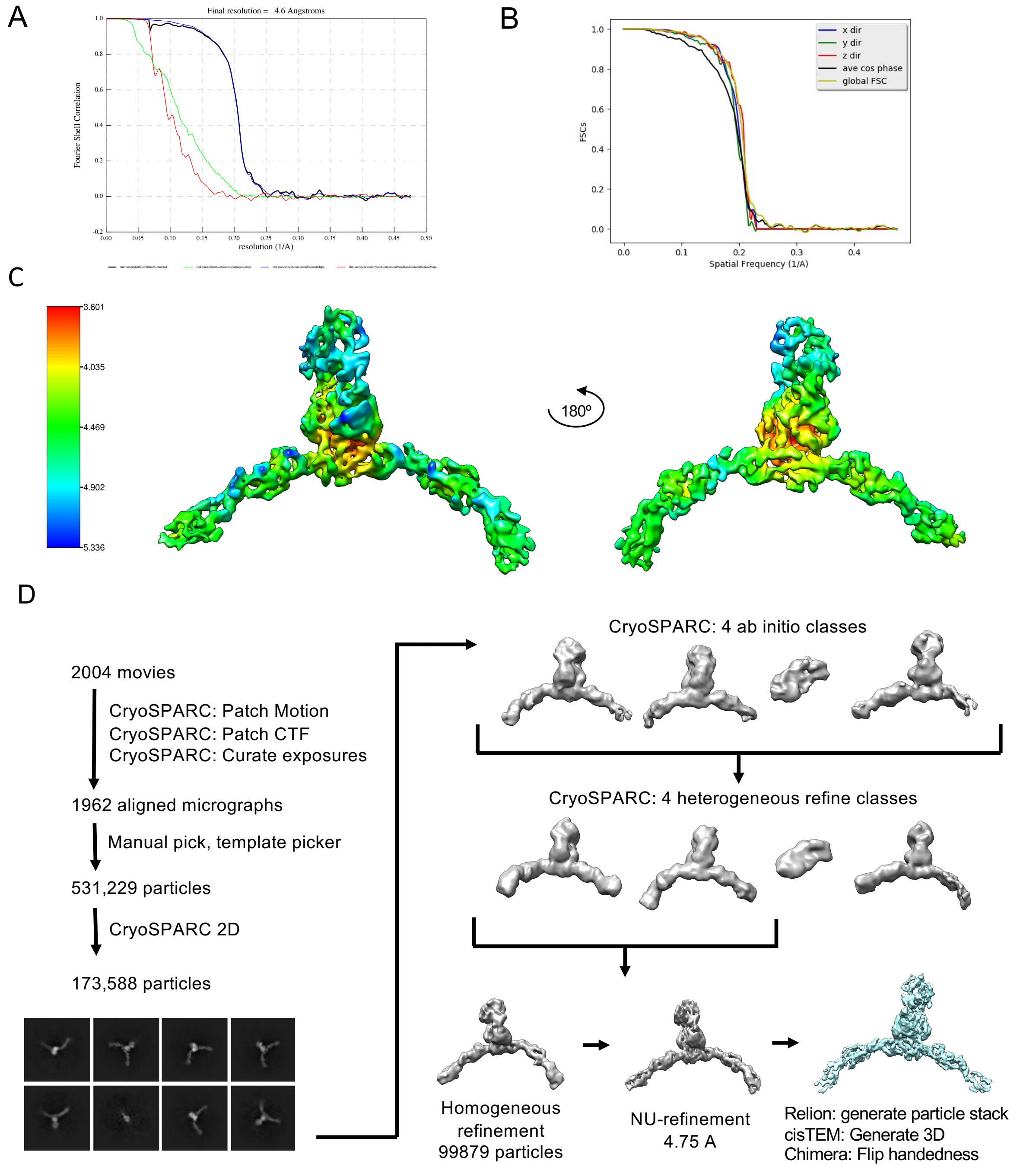

Figure S10. Cryo-EM characterization of FL-hE-cadherin + 46H7Fab. **(A)** Relion Gold-Standard 0.143 FSC resolution of final reconstruction. **(B)** Directional resolution of map calculated by 3DFSC server. Calculated sphericity: 0.982. **(C)** Local resolution estimation over sharpened final 3D reconstruction. **(D)** Data processing pipeline toward final reconstruction.

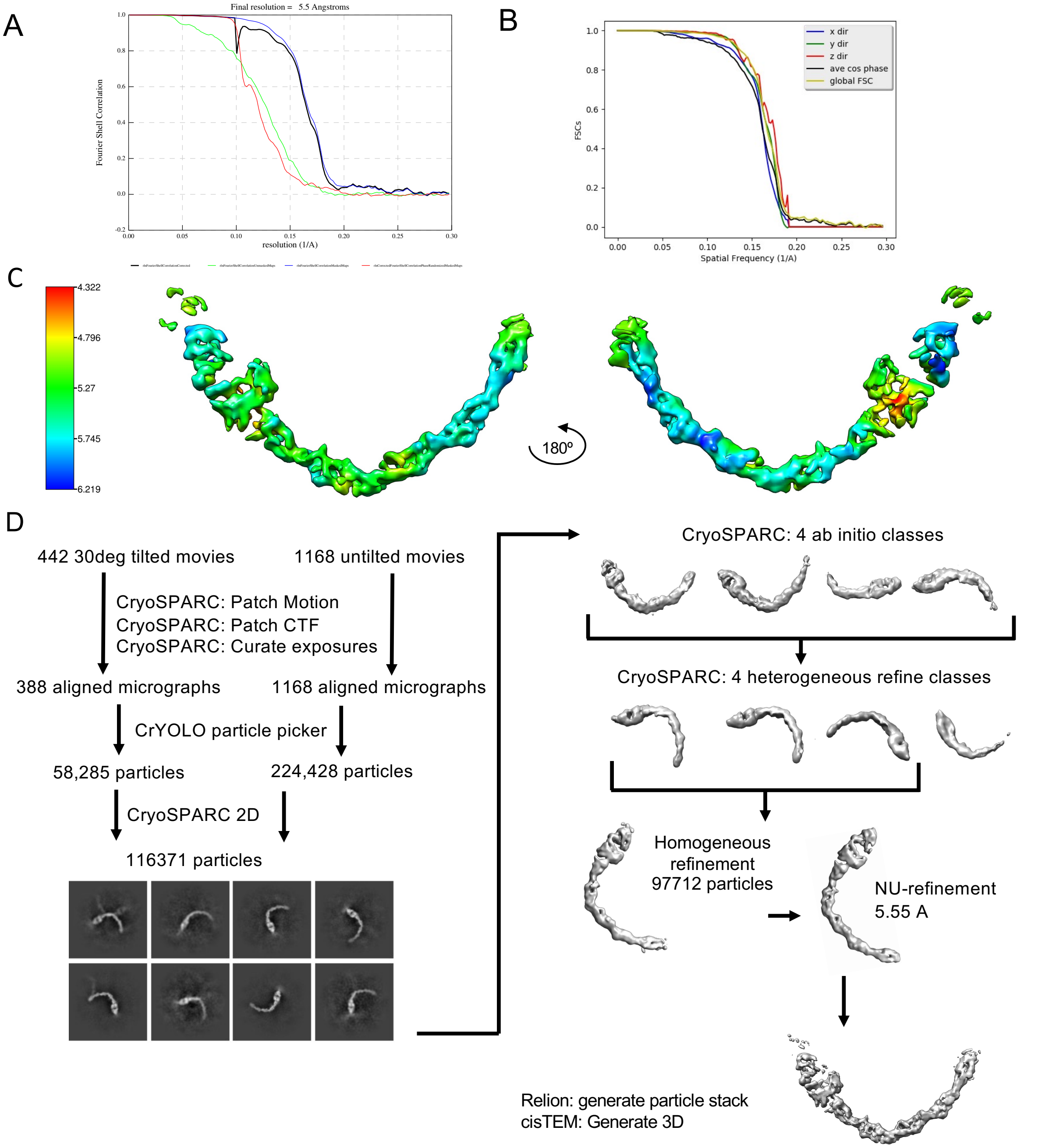

Figure S11. Cryo-EM characterization of FL-hE-cadherin + 67G8Fab. **(A)** Relion Gold-Standard 0.143 FSC resolution of final reconstruction. **(B)** Directional resolution of map calculated by 3DFSC server. Calculated sphericity: 0.963. **(C)** Local resolution estimation over sharpened final 3D reconstruction. **(D)** Data processing pipeline toward final reconstruction.
